# In Pursuit of the Epithelial Mechanosensitivity Mechanisms

**DOI:** 10.3389/fendo.2018.00804

**Published:** 2019-01-15

**Authors:** Arthur Beyder

**Affiliations:** ^1^Enteric Neuroscience Program, Division of Gastroenterology and Hepatology, Department of Medicine, Mayo Clinic, Rochester, MN, United States; ^2^Department of Physiology and Biomedical Engineering, Mayo Clinic, Rochester, MN, United States

**Keywords:** enteroendocine cells, mechanosensitivity, mechanosensitive ion channel, serotonin, enteric nervous system

## Abstract

Mechanosensation is critical for normal gastrointestinal (GI) function. Disruption in GI mechanosensation leads to human diseases. Mechanical forces in the GI tract are sensed by specialized mechanosensory cells, as well as non-specialized mechanosensors, like smooth muscle cells. Together, these cellular mechanosensors orchestrate physiologic responses. GI epithelium is at the interface of the body and the environment. It encounters a variety of mechanical forces that range from shear stress due to flow of luminal contents to extrinsic compression due to smooth muscle contraction. Mechanical forces applied to the GI mucosa lead to a large outflow of serotonin, and since serotonin is concentrated in a single type of an epithelial cell, called enterochromaffin cell (ECC), it was assumed that ECC is mechanosensitive. Recent studies show that a subset of ECCs is indeed mechanosensitive and that Piezo2 mechanosensitive ion channels are necessary for coupling force to serotonin release. This review aims to place this mechanism into the larger context of ECC mechanotransduction.

The gastrointestinal (GI) tract is responsible for sensing luminal chemical and mechanical stimuli (Figure 1A) to coordinate the processes of digestion and absorption of ingested nutrients and excretion of wastes. The GI tract also sends signals to the rest of the organism regarding the composition of the luminal contents during digestion, to prepare the metabolic and cardiovascular systems for the flood of absorbed chemicals, and also signals about the lack of nutrients during fasting to assist in adjusting metabolic mechanisms (1). To accomplish these tasks, the lining of the GI tract developed a repertoire of specialized epithelial sensors called enteroendocrine cells (EECs) (2). These cells are distributed sporadically throughout the entire GI tract and serve as beacons of luminal signaling. They sense the nutrients and mechanical stimuli and convert them into physiologically meaningful responses—via secretion of hormones, and local signaling with the intrinsic and extrinsic nerves (3, 4). In turn, EEC disruptions contribute to human diseases that range from gut-centric, such as diarrhea and constipation, to gut-brain axis, such as irritable bowel syndrome (IBS), and system-wide, such as obesity (5).

**Figure 1:**
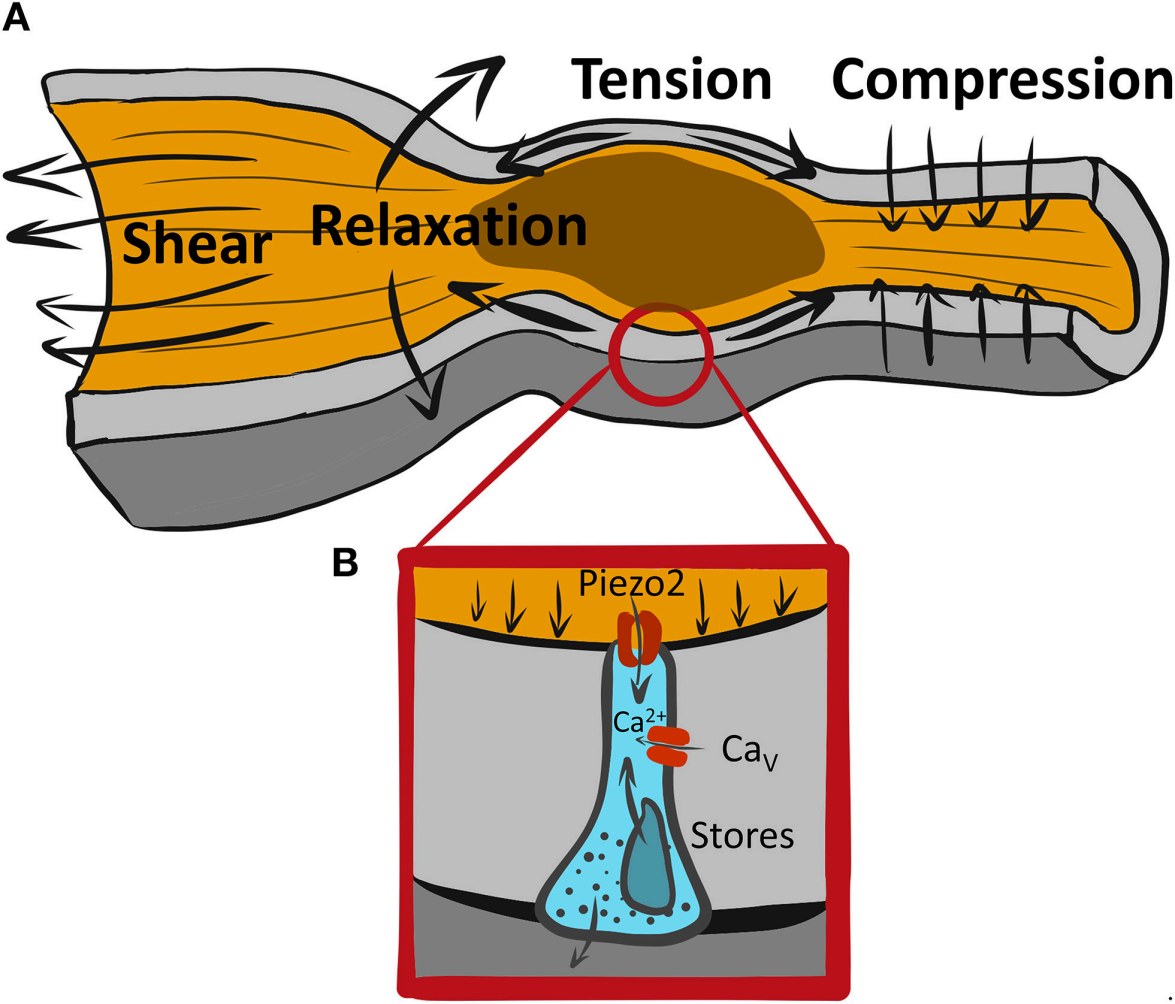
A variety of mechanical forces associated with GI motility may be sensed by mechanosensitive EECs. **(A)** An example of a range of forces associated with propulsive GI motility due to proximal (oral) contraction and distal (aboral) relaxation leading to tension at the wall and shear stress from flowing luminal contents. **(B)** Piezo2+ mechanosensitive EEC senses forces via Piezo2 mechanosensitive ion channels, which produce a “receptor current” leading to intracellular Ca^2+^ increase possibly due amplification by membrane signaling via voltage gated calcium channels (Ca_*v*_s) and Ca^2+^ release from intracellular stores.

One of the enteroendocrine cell types, called the enterochromaffin cell (ECC), synthesizes majority of peripheral serotonin (5-hydroxytryptamine, 5-HT). ECC 5-HT has a range of important local effects, like regulation of GI motility and secretion (3, 6), and systemic effects on metabolism during fasting (1). Like other EECs, ECCs are activated by luminal chemical stimuli (7). However, these cells appeared to be different from other EECs when Edith Bülbring found that mechanical pressure applied to the epithelium resulted in release of 5-HT, suggesting that ECCs may also be mechanosensitive (8).

ECC mechanosensitivity was inferred from Dr. Bülbring's experiments that connected luminal pressure to 5-HT release, and has since been demonstrated in other studies in animals (9) and humans (10). However, since the enteric nervous system has complex multicellular organization, and multiple cell types are mechanosensitive, it was important to determine whether ECCs are intrinsically mechanosensitive, or if they respond to signals from other mechanosensitive cells in the GI tract.

## Mechanosensitivity of Immortalized 5-HT Secreting Neuroendocrine Cells

ECCs make up ~1% of the epithelium, and their random distribution makes them difficult to purify and identify. Further, epithelial cells are constantly turned over in a balanced process of stem cell replication and anoikis, or attachment dependent apoptosis. Therefore, isolated epithelial cells have very short lifespans, so primary cultures of ECCs have a limited shelf life. Because of these issues, first studies that examined ECC mechanosensitivity used immortalized 5-HT secreting cells from neuroendocrine tumors (11). A pancreatic neuroendocrine cell, called BON, was cultured and mechanically stimulated by rotational shaking, which resulted in 5-HT release (12). This 5-HT release depended on intracellular Ca^2+^ increase that was driven by activation of G_α*q*_ leading to release of Ca^2+^ from intracellular stores. While G_α*q*_ is not known to be mechanosensitive, G_α*q*_ activation in BON cells required association with caveolins, which are mechanosensitive (13). Mechanical stimulation of BON cells by touching or rotational shaking also led to ATP release and autocrine activation of P2X and P2Y receptors (14). A follow up study showed involvement of adenosine receptors in BON cell mechanotransduction (15), and mechanotransduction of a different cancer cell line, KRJ-I (16).

Specialized epithelial mechanosensors are distributed throughout our bodies, and include Merkel cells in the skin required for touch (17) and hair cells in the ear required for hearing (18). Recent work showed that Merkel cells rely on a mechanosensitive ion channel called Piezo2 (*Piezo* is Greek for “squeeze” or “press”). There are developmental and functional similarities between Merkel and ECCs—they have multiple common developmental transcription factors, are both mechanosensitive and secrete 5-HT (19). So, we examined whether Piezo2 channels may contribute to ECC mechanosensitivity. We used another cancer-derived 5-HT secreting neuroendocrine cell, called QGP-1, and found that Piezo2 was expressed (20). When these cells were mechanically stimulated by direct membrane displacement, they produced a force-induced ionic current that had biophysical properties of Piezo2—rapid onset and inactivation, and steep mechanosensitivity without rectification. Further, when we grew QGP-1 cells on flexible membranes and stretched them, we found that they released 5-HT. This mechanosensitive 5-HT release was inhibited by a variety of mechanosensitive and Piezo ion channel blockers and importantly by Piezo2 siRNA but not non-targeted (NT) siRNA (20). In all, studies using immortalized neuroendocrine cell lines have and continue to provide valuable information on EEC mechanosensitivity (11). The data in 5-HT releasing neuroendocrine cell lines showed that they are mechanosensitive and that they employ a range of mechanisms of mechanotransduction to convert force into 5-HT release—including G-protein coupled pathways as well as ionic pathways. The results on Piezo2 ion channel were particularly intriguing to us, because this ion channel is established to be mechanosensitive (21), unlike the other described molecules, and it was shown to be a primary mechanosensor critical for mechanosensitivity in other epithelial mechanosensors.

## Mechanosensitivity of Primary ECCs

### What Is the Primary Mechanosensor?

A critical question is whether primary ECCs are mechanosensitive. Chin et al. purified ECCs from patients with inflammatory bowel disease and grew them on flexible substrates and when they stretched them, they found 5-HT release that depended on adenosine signaling (16). However, the nature of the mechanosensor upstream of this mechanism remained unclear. We pursued the hypothesis that Piezo2 channels were important for ECC mechanosensitivity (20, 22, 23). We examined Piezo2 expression in human jejunum and transgenic reporter and lineage traced mice and found that Piezo2 was expressed in ECCs but that not all ECCs expressed Piezo2 (20, 22). Further, while majority of Piezo2 was present in 5-HT positive cells, some Piezo2+ cells were 5-HT negative (22). So, we explored whether EECs were mechanically sensitive (22). Using electrophysiology, we found that more than 50% of EECs had mechanosensitive ionic currents, unlike other, likely epithelial cells, in primary culture. Membrane displacement produced fast ionic currents—they activated with rapid membrane displacement within milliseconds and inactivated almost as rapidly—within few dozen milliseconds. EEC mechanosensitive currents were steeply mechanosensitive, going from off to on within 2 μm membrane displacement, and they were non-rectifying. These were biophysical properties of Piezo2 channels, so we used pharmacology and knockdown to determine whether the EEC mechanosensitive currents were indeed carried by Piezo2. However, given the speed of the mechanosensitive currents, we wondered how those fast currents could lead to 5-HT release that lasts for seconds? Using Ca^2+^ imaging, we found that in isolated mechanosensitive EECs both shear stress and membrane displacement led to a fast rise in intracellular Ca^2+^ but return to baseline Ca^2+^ took tens of seconds, regardless of whether ECCs were stimulated very briefly (50 ms) or more slowly (20 s). Mechanosensitive increase in Ca^2+^ was dependent on Piezo2 and was required for 5-HT release, which we measured in single cells using biosensors. As with intracellular Ca^2+^ increase, mechanically stimulated 5-HT release lasted for several seconds after even one rapid (50 ms) stimulation.

## There are Many Remaining Questions

### What Are the Mechanotransduction Pathways That Link Piezo2 and 5-HT Release?

Considering how fast Piezo2 inactivates, the duration of Ca^2+^ increase with mechanical force is interesting, but not surprising. Amplification of the rapid “receptor current,” such as Piezo2, is common in sensory neurobiology, and it occurs in other epithelial mechanosensors, such as Merkel cells and hair cells in the ear (24) (Figure 1B). Cells frequently use Ca^2+^ signaling to regulate the amplitude and duration of the response to receptor current. Though it is currently unclear, Piezo2 receptor current, which is non-selective for Na^+^ and Ca^2+^, may have three possible roles: (1) it could bring in some Ca^2+^ which would initiate 5-HT release that may stimulate further secretion by autocrine mechanism, (2) it could depolarize the ECC membrane and lead to activation of sodium or calcium voltage-gated channels (7, 25, 26), and/or (3) it could bring in Ca^2+^ which may activate Ca^2+^ activated Ca^2+^ release. Ca^2+^ handling mechanisms are important in ECC function, both in the context of chemo- and mechano-sensation. Thus, several types of voltage-gated Ca^2+^ channels are found by expression and functional analysis using pharmacologic blockers and voltage-clamp in both immortalized neuroendocrine cells and primary EECs. These include L-type (Ca_V_1.3, Cacna1d), T-type (Ca_V_3.2, Cacna1h), and P/Q-type (Ca_V_2.1, Cacna1a) (7, 26–28). Both L-type (26) and P/Q-type (7) channels, but interestingly not the highly expressed T-type Ca_V_ channels, have found their functional relevance in chemotransduction, but not all studies agree on the roles of these channels in chemotransduction. Future work will need to determine whether Ca_V_ channels are involved in ECC mechanotransduction.

### Are Human EECs Mechanosensitive?

As I describe above, multiple studies examined mechanosensitivity of human immortalized neuroendocrine cells. But we still have limited knowledge about human enteroendocrine cell mechanosensitivity. We know that mechanical stimulation of ECCs leads to 5-HT release (16), but we do not know whether increased 5-HT is due to increased secretion or decreased reuptake (10). Purified human ECCs from patients with inflammatory bowel disease were grown in primary culture on flexible substrates lead to stretch-dependent 5-HT release that depend on activation of ADORAs (16). However, since adenosine and ATP are frequently released by mechanical stimuli by non-specialized mechanosensitive cells (29), we do not know whether 5-HT release from these cells is due to their being specialized mechanosensors. So, while Piezo2 channels were found in human jejunum ECCs (20), but we do not know whether these cells are mechanosensitive. Progress in the field of human ECC physiology is hampered by the same barriers that limited the studies of ECCs from animal models—they are sparse and primary cultures do not survive long term. Yet, significant progress is being made in human epithelial cell models and culturing techniques, suggesting that intellectual progress on human EEC physiology is not far behind.

### Are ECCs the Only Mechanosensitive EECs?

A recent study in drosophila showed that a population of EEC precursors express Piezo channels (drosophila has only one Piezo gene) and regulate the density of mechanosensitive EECs which are important to respond to luminal filling or compression due to muscle contractions (30). Our recent work shows that Piezo2 is mostly in ECCs, but both by immunohistochemistry and 5-HT release measurements, not all mechanosensitive EECs are ECCs (22). This is not surprising, as recent studies show that delineation between different EEC subtypes that we are used to is less accurate than seeing ECCs as a part of a continuous EEC spectrum, suggesting that the differences between EEC subtypes may be subtle in mice (31) and humans (32). Further, EEC phenotype is not stable, since expression of both signaling molecules and receptors varies along with cell migration through the crypt-villus axis (33). These circumstantial clues suggest that mechanosensitive ECCs may release bioactive substances along with 5-HT, and in addition to ECCs, other EECs may be mechanosensitive, and finally that mechanotransduction elements may be differentially expressed during EEC development. For us to understand EEC roles in physiology, we first must understand the repertoire of mechanosensitive EECs and their products.

### What Are the Physiologically Relevant Forces in the Epithelium?

Studies aiming to understand GI mechanotransduction at the single cell level use a variety of mechanostimulation techniques. Some notable examples include shear stress (34), direct membrane displacement by probes (20, 22) rotational shaking (12), pressure clamp (34), and stretch of flexible substrates (16, 20). Each of these techniques has its advantages and disadvantages, so it is important to use multiple techniques on the same preparation. Single EEC mechanosensitivity was tested using rotational shaking (12), membrane displacement (22), and stretch via flexible membranes (16, 20), and these stimuli produced responses, as measured by 5-HT, intracellular Ca^2+^ and membrane currents as read outs. However, it is not always clear what forces the mechanosensitive cells encounter (Figure 1A). Specialized mechanosensitive cells are built to respond to acute mechanical stimuli, but they also reside in an environment that has resting mechanical forces. The gut wall is a composite material with different layers (mucosa, submucosa, muscularis, and serosa) having different mechanical properties. At the tissue scale, mucosa has non-trivial resting mechanical energy, which is placed within the confines of a mechanically stiff muscularis layer (35). The situation is no less complex within the epithelium. For example, during the peristaltic reflex (Figure 1A) an epithelial cell, such as EEC, likely encounters several different forces. It feels the compressive force from proximal muscle contraction against luminal contents, stretch due to distal relaxation and shear stress from the flow of luminal contents. This means that an ECC at the tip of the villus likely experiences shear and compression, while an ECC in the crypt experiences compression and stretch, but much less shear stress. At the cellular scale, EECs reside within an epithelial monolayer, which is a crowded setting, and the resting forces that exist due to crowding (36), and on a larger scale, the villi have a resting stiffness, which provides background mechanical force for the epithelium (35). So, acute forces from GI physiologic processes need to be detected from above the resting mechanical background. To make progress, we need to understand not just the mechanisms of EEC mechanotransduction, but also the mechanical environment within which physiologic forces exist. Further, since there are several mechanosensitive cell types in the GI tract which are arranged in complex mechanosensory circuits, the nature and location of force to each of the mechanosensors is integrated to obtain physiologic effect. For example, do ECCs respond to luminal forces, such as secretion-driven volume expansion and shear stress, or to muscularis driven contraction compressing the mucosa, or both?

In conclusion, recent advances in ECC physiology and mechanosensitivity have uncovered specific mechanotransduction pathways that couple GI forces to 5-HT release. This progress will lead to better understanding of ECC contributions to GI physiology, and whether ECC mechanosensation contributes to GI pathophysiology. However, many important questions remain, including understanding of the specifics of mechanism of mechanotransduction in animal models and humans, the repertoire of mechanosensitive EECCs, and how ECC mechanosensitivity fits into the context of GI mechanobiology.

## Author Contributions

AB planned, wrote, and edited the paper.

### Conflict of Interest Statement

The author declares that the research was conducted in the absence of any commercial or financial relationships that could be construed as a potential conflict of interest.
